# Broadening of attention dilates the pupil

**DOI:** 10.3758/s13414-023-02793-3

**Published:** 2023-10-06

**Authors:** Martin Kolnes, Andero Uusberg, Sander Nieuwenhuis

**Affiliations:** 1 Department of Psychology Institute of Psychology, University of Tartu, Näituse 2, 50409 Tartu, Estonia; 2 Institute of Psychology and Leiden Institute for Brain and Cognition, Leiden University, Leiden, The Netherlands

**Keywords:** Pupil dynamics, Breadth of attention, Visual attention

## Abstract

Inconclusive evidence suggests that the pupil is more dilated when the breadth of attention is broad compared to narrow. To further investigate this relationship, we recorded pupil size from healthy volunteers while inducing trial-wise changes in breadth of attention using a shape-discrimination task where participants had to remember the location of a gap in a small or a large circle. A visual search task with targets presented at different distances from the centre of the screen was used to behaviourally assess the success of the manipulation of breadth of attention. Data were analysed using a generalised additive mixed model to test the experimental effects on pupil size after controlling for the effects of gaze location and eye vergence. The results showed that the pupil was more dilated in the broad-breadth-of-attention condition compared to the narrow-breadth-of-attention condition. However, the effect of attentional breadth on visual search performance was not mediated by pupil size, suggesting that more research is needed to understand the functional role of pupil dilation in relation to breadth of attention.

**Supplementary Information:**

The online version contains supplementary material available at 10.3758/s13414-023-02793-3.

## Introduction

At any given time, our visual system is bombarded by a rich stream of sensory inputs that compete for our attention. To avoid being overburdened by information, we focus on only a few stimuli at a time, using our metaphorical spotlight of attention to prioritize the processing of some areas of the visual field at the expense of others (Posner et al., 1980). In addition to controlling the spatial location of the spotlight, attention regulation also involves controlling the size of the spotlight – that is, the breadth of attention. Thus, a perhaps more appropriate metaphor for attention is that of a zoom-lens that can narrow or widen the focus of attention (Eriksen & James, 1986). This metaphor is consistent with findings that a narrow breadth of attention enhances the perception of fine detail in a small portion of the visual field, whereas a wide breadth of attention prioritizes a larger area at the expense of perceptual resolution (Eriksen & James, 1986; Lawrence et al., 2020). Next to known neural correlates of breadth of attention (Müller et al., 2003), there is recent but inconclusive evidence suggesting that attention breadth may also correlate with pupil size (Mathôt, 2020). Furthermore, it has been suggested that pupil responses have a functional role by adjusting vision to the demands of narrow versus broad breadth of information processing (Mathôt, 2020). The current study was designed to test the hypothesis that pupil size scales with the breadth of attention. We also investigated whether pupil size mediates the behavioural effects of breadth of attention.

The main function of pupillary reactivity is to regulate the amount of light that enters the eye. This regulation mechanism is mainly demonstrated by the pupillary light reflex – a rapid change in pupil size caused by changes in light conditions (Ellis, 1981). Additionally, pupil size is influenced by a wide variety of cognitive factors (Beatty, 1982; Mathôt, 2018), including working memory load (Kahneman & Beatty, 1966), mental calculation (Ahern & Beatty, 1979), and emotional arousal (Bradley et al., 2008). Recently, it has been suggested that breadth of attention may be another cognitive factor that influences pupil size (Mathôt, 2020).

There are both conceptual and empirical reasons to hypothesize that pupil size and breadth of attention may be correlated. On a conceptual level, both pupil size and breadth of attention optimize early visual processing in a similar manner. On the one hand, the size of the pupil influences the trade-off between visual acuity and sensitivity (Mathôt & Van der Stigchel, 2015). The dilation of the pupil blurs the retinal image, but increases overall visual sensitivity, while constriction of the pupil sharpens the retinal image, but decreases overall visual sensitivity (Daniels et al., 2012; Mathôt & Van der Stigchel, 2015). These divergent consequences of dilated and constricted pupils most likely stem from different amounts of light reaching the eye, as well as from different spherical aberration – changes in the eye lens that influence the refraction of light rays through the lens (Campbell & Gubisch, 1966; Koomen et al., 1949). On the other hand, breadth of attention has also been linked to early visual processing. A narrow breadth of attention increases visual acuity for the small attended area (Balz & Hock, 1997), while a broad breadth of attention allows for  the processing of a wider area, but decreases overall visual acuity (Eriksen & James, 1986; Lawrence et al., 2020). Given their similar functions, it is possible that smaller pupil size co-occurs with narrower breadth of attention while larger pupil size co-occurs with broader breadth of attention.

On an empirical level, there is also preliminary evidence that changes in pupil size coincide with changes in attentional breadth (Brocher et al., 2018; Daniels et al., 2012; DiCriscio et al., 2018; Eldar et al., 2016; Mathôt & Ivanov, 2019). Daniels et al. (2012) instructed participants to shift their focus of attention between centrally presented stimuli (radius of 1.3°) and peripherally presented stimuli (radius of 13°), as indicated by rhythmic changes in the colour of a central fixation dot. They found that these rhythmic alternations in the breadth of attention were reflected in the magnitude of pupil oscillations at the attended frequency. In a more traditional study, Brocher et al. (2018) instructed participants to covertly attend to stimuli presented one at a time at various distances from a central fixation point (varying from 12.5° to 42.5°). The study showed that the pupil dilation response to these stimuli increased gradually with increasing distance from the fixation point. DiCriscio et al. (2018) manipulated breadth of attention by instructing participants to attend to either the local or global elements of hierarchical stimuli (e.g., a large letter “P” made up of small letters “H”). They found that the pupil response to these stimuli was larger during the selection of global information compared to local information. Finally, Mathôt and Ivanov (2019) directly manipulated pupil size by varying peripheral brightness. They showed that small pupils are more beneficial in tasks that require central visual acuity, while large pupils are more beneficial in tasks that require detection of faint stimuli in peripheral vision. Although methodologically quite diverse, these studies all support the notion that the pupils dilate in response to a widening of attentional focus.

At the same time, the evidence for this idea is still inconclusive. In addition to the relatively small number of existing studies, there are two important concerns that relate not just to these studies, but to this research question in general. First, covertly attending to stimuli in the peripheral visual field can require more mental effort than attending to stimuli in the central visual field (Brocher et al., 2018; Hüttermann & Memmert, 2017). As mental effort is a known driver of pupil dilation (van der Wel & van Steenbergen, 2018), it is possible that the difference in pupil size between narrow and broad breadth of attention observed in prior studies stems at least in part from the difference in required mental effort rather than from the effect of attentional breadth per se. The effect of mental effort may explain some existing findings (Brocher et al., 2018), although not all (Daniels et al., 2012; Mathôt & Ivanov, 2019). In the present study, we sought to minimize the potential confounding effect of mental effort by investigating the relationship between attention breadth and pupil size using stimuli that fell broadly within the centre of the visual field, the central area with a radius of 8 degrees of visual angle (Strasburger et al., 2011).

The second concern with existing studies is that inducing a narrow or wide breadth of attention can induce convergence and divergence eye movements, respectively. These eye movements are connected to the pupil near response: the constriction of the pupil in response to looking at nearby objects, and the dilation of the pupil in response to looking at distant objects (Mathôt, 2018). Therefore, convergence and divergence eye movements can become additional sources of confounding variance in pupil size measurements. In the present study, we sought to statistically control for the potential confounding effects of eye movements.

In addition to replicating prior findings with improved control over confounds, more research is needed to understand the functional significance of the correlation between pupil size and breadth of attention. One possibility is that changing pupil size is a mechanism through which attention control systems achieve the narrowing and broadening of the focus of attention. Specifically, as mentioned above, the size of the pupil may influence the trade-off between visual acuity and sensitivity: a constricted pupil is thought to be associated with reduced optical blur and high visual acuity in foveal vision, while a dilated pupil allows more light to enter the eye, resulting in increased visual sensitivity in peripheral vision (Mathôt & Van der Stigchel, 2015). Under this assumption, it would be expected that variation in pupil size partially mediates the effects of experimental manipulations of breadth of attention on behaviour. In this study, we conducted a statistical mediation analysis to test this hypothesis. However, given the limited empirical support for this proposition, it is also possible that the relationship between pupil size and breadth of attention is merely correlational. That is, while pupil size may reflect the activity of the mechanisms that impact breadth of attention, it may not contribute to the breadth-of-attention effect on perception. For instance, pupil size and breadth of attention may be independently influenced by the activity of the central noradrenergic system. Pupil size has been shown to reflect the activity of this system (Aston-Jones & Cohen, 2005; Joshi & Gold, 2020). At the same time, the noradrenergic system has been implicated in regulating exploration-exploitation modes of behaviour, and the breadth of attention could be modulated in the service of exploration and exploitation of environmental opportunities, respectively (Aston-Jones & Cohen, 2005; Gable & Harmon-Jones, 2010).

### Present study

Although previous studies suggest that pupil size scales with the breadth of attention, these findings await replication in concert with efforts to minimize potential sources of confounding variance such as mental effort and the pupil near response. The present study was designed to meet these aims. To this end, we devised a novel experimental paradigm in order to expand the set of conditions under which the link between attention breadth and pupil size has been investigated. By presenting all stimuli within the central area of the visual field (radius of 7°), we sought to limit the potential confounding effects of more mental effort being required to detect stimuli in the peripheral visual field. The relatively central location of stimuli also served to limit convergence and divergence eye movements that are a part of the pupil near response (Mathôt, 2018). In addition, to analyse the pupil data we used a generalised additive mixed model (Hastie & Tibshirani, 1990; van Rij et al., 2019; Wood, 2017) that allowed us to statistically control for the effects on pupil size of gaze location (Hayes & Petrov, 2016) and convergence eye movements.

**Fig. 1:**
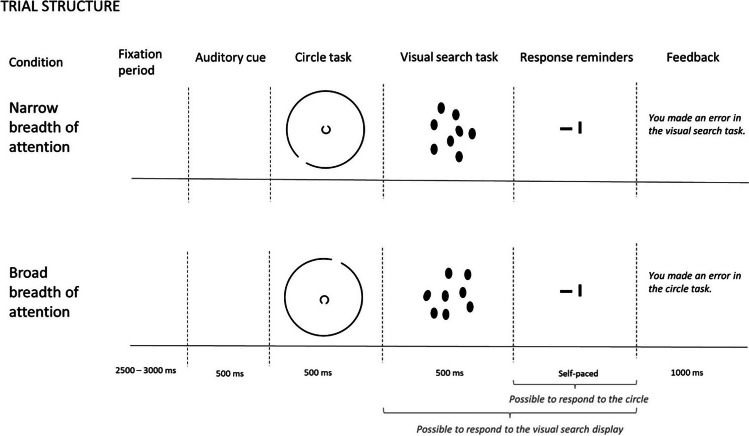
Trial structure. When the participant had already responded to the target ellipse during the presentation of the visual search display, only the response reminder for the circle task was presented. Feedback was presented only when the participant made an error in one or both of the tasks

We combined a shape-discrimination task as an attentional breadth induction procedure (Goodhew et al., 2016) with a visual search task as a manipulation check (Fig. 1). In the shape-discrimination task, participants needed to detect the location of a gap in a Landolt circle (Kliegl et al., 2015). Small Landolt circles were used to induce a narrow breadth of attention and large Landolt circles were used to induce a broad breadth of attention (Goodhew et al., 2016; Ronconi et al., 2014). We hypothesized that the pupil would be smaller in the narrow-breadth-of-attention condition than in the broad-breadth-of-attention condition. Additionally, we used a visual search task in which participants had to detect one tilted ellipse (the target) among eight distractor ellipses. We manipulated the vertical or horizontal distance of the target ellipse from the centre of the screen. Finally, we examined if variation in pupil size would mediate the effect of breadth of attention manipulation on reaction times (RTs) in the visual search task.

To analyse performance on the visual search task, we used a drift diffusion model (DDM) which translates reaction-time data and error rates into a number of latent decision-making parameters and thus allows an unambiguous quantification of performance differences between task conditions (Ratcliff, 1978). The DDM assumes that during decision making information is accumulated over time in favour of one of two choice alternatives, and a decision is made when a decision boundary is reached for one of the alternatives (Ratcliff & McKoon, 2008). The DDM is designed to disentangle different response strategies in simple two-choice RT tasks that might confound standard analyses of RTs and error rates (Ratcliff & McKoon, 2008; Voss et al., 2013). Although the task in our experiment is more difficult than a simple two-choice task, the DDM has also been used for tasks with high error rates and slow (>1 s) responses (e.g., McGovern et al., 2018). We were mainly interested in the drift-rate parameter, which reflects the perceptual processing efficiency of the participant (Voss et al., 2013). The higher the drift rate the more quickly information is accumulated towards one decision over the other. A narrow breadth of attention should enhance information accumulation for targets near the centre of the screen, while a broad breadth of attention should enhance information accumulation for targets farther away from the centre of the screen.

Overall, we expected performance in the visual search task to decrease with increasing distance of the target from the centre of the screen, as reflected in a decrease in drift rate. Importantly, as a manipulation check, we also expected this performance decrease to be milder in the broad- compared to narrow-breadth-of-attention condition.

## Methods

### Participants

In total, 24 participants completed the study. This sample size was based on a pilot study with 21 participants that showed a significant effect of our breadth-of-attention manipulation on performance in the visual search task). Two participants were excluded after pre-processing of the reaction time data (see *Pre-processing of behavioural data*), resulting in a final sample of 22 participants (four males, age: *M* = 25.7, *SD* = 5.8 years). Participants were university students recruited through community and campus mailing lists. Psychology students received 1.5 course credits for taking part in the experiment. Exclusion criteria included presence of neurological or psychiatric disorders. Participants were asked to avoid alcohol or other drugs 24 h prior to the experiment, and not to drink coffee 3 h prior to the experiment. All participants provided written informed consent. The study was approved by the Institutional Review Board of University of Tartu.

### Design and procedure

Participants were seated behind a table with a headrest fixed at 66 cm from the computer monitor. The experiment combined two tasks: a shape-discrimination task (circle task) that served as an induction procedure (Goodhew et al., 2016) and a visual search task (ellipse task) that served as a behavioural manipulation check (Fig. 1). The first task was used to induce a narrow or wide breadth of attention. The second task was used to measure the effect of the induced attentional breadth on perception. Before the experiment, participants received written instructions about the two tasks and were asked to keep their gaze on the middle of the screen throughout the experiment. In addition, participants completed eight practice trials.

Each trial started with a blank screen for 2,500–3,000 ms, drawn randomly from a uniform distribution. This intertrial interval allowed the pupil to return to baseline before the start of the 500-ms baseline period used to correct the pupil dilation response on the subsequent trial. Next, an auditory cue, a 500-ms tone of variable pitch, signalled whether in the current trial the participant should focus on the small circle (440 Hz) or the large circle (294 Hz). To minimize the potential confounding effect of tone pitch on our results, we took two measures. First, we chose tone cues that had only a small difference in pitch. Second, we paired the higher pitch cue with the narrow condition and the lower pitch cue with the broad condition. Because higher-pitched tones tend to dilate the pupil more than lower-pitched tones (Liao et al., 2016), we made sure that this potential nuisance effect worked *against* our hypothesized effect of larger dilation in the broad breadth-of-attention condition, and therefore could not present an alternative explanation of the expected results.

Next, the circle task started with the 500-ms presentation of two Landolt circles of small (radius of 0.7°) and large (radius of 7°) size (Kliegl et al., 2015). The small circle was expected to induce a narrow breadth of attention, while the large circle was expected to induce a broad breadth of attention. To make sure participants paid attention to the cued circle, they were asked to remember the location of the gap in that circle: downward facing or upward facing.

Next, the visual search display was presented for 500 ms. Participants had to detect among eight ellipses one that was tilted to the left or right (the target ellipse). The target ellipse appeared at one of three fixed distances from the centre of the screen. The participants were instructed to respond to the target ellipse as quickly as possible while making as few mistakes as possible.

Finally, one or two response reminders were shown on the screen. The first response reminder was a horizontal line that cued the participant to indicate via a key press with their right hand whether the target ellipse was tilted to the left or to the right side. The second response reminder was a vertical line which instructed the participant to indicate whether the gap in the cued circle was facing upwards or downwards. If the participant had already responded to the target ellipse during the presentation of the visual search display, then only the second response reminder was shown. If the participant made an incorrect response in one or both of the tasks, the trial ended with an error message, presented on the screen for 1 s.

The session began with a 9-point calibration procedure of the eye-tracker. Before the start of the experiment, participants were also encouraged to listen to the auditory cues until they were confident that they memorized the meaning of the cues. To make sure that the link between the auditory cues and the circles was correctly memorized, participants were also able to listen to the cues at the beginning of each experiment block.

The experiment consisted of eight blocks of 48 trials. The sequence of the trials was randomized in each block. Participants were then asked to take a small pause before continuing with the next block. After the fourth block, participants were asked to rest, stretch, and play a 2-min browser game Jewelish (*Jewelish*, n.d.). After the pause, the eye-tracking calibration procedure was repeated, and the participants completed the final 4 blocks of trials. In total, each participant completed 384 trials: 64 trials for each of the six factorial combinations of circle size (large or small) and target distance from the centre of the screen (1°, 3° or 6°).

### Stimuli and apparatus

Stimuli were presented on a grey background of a gamma-corrected monitor (19-in. Dell E1911; 1,024 × 768 px) in a dimly lit room (6 lux). The red colour of the circle in the circle task was isoluminant with the background (6.7 cd/m^2^). The small circle, used to induce a narrow breadth of attention, was a circle outline with a radius of 0.7° of visual angle. The large circle, used to induce a broad breadth of attention, was a circle outline with a radius of 7° of visual angle. The gap size was 0.45° (11 participants) or 0.60° (13 participants) of visual angle in the small circle, and 3° of visual angle in the large circle. We increased the gap in the small circle after 11 participants in an attempt to match the difficulty of gap detection in the large and small circles. On half of the trials the gap location was facing downward, on the other half it was facing upward. The exact location of the gap was varied randomly across trials between 315° and 45° for the upward facing gap, and between 135° and 225° for the downward facing gap.

**Fig. 2:**
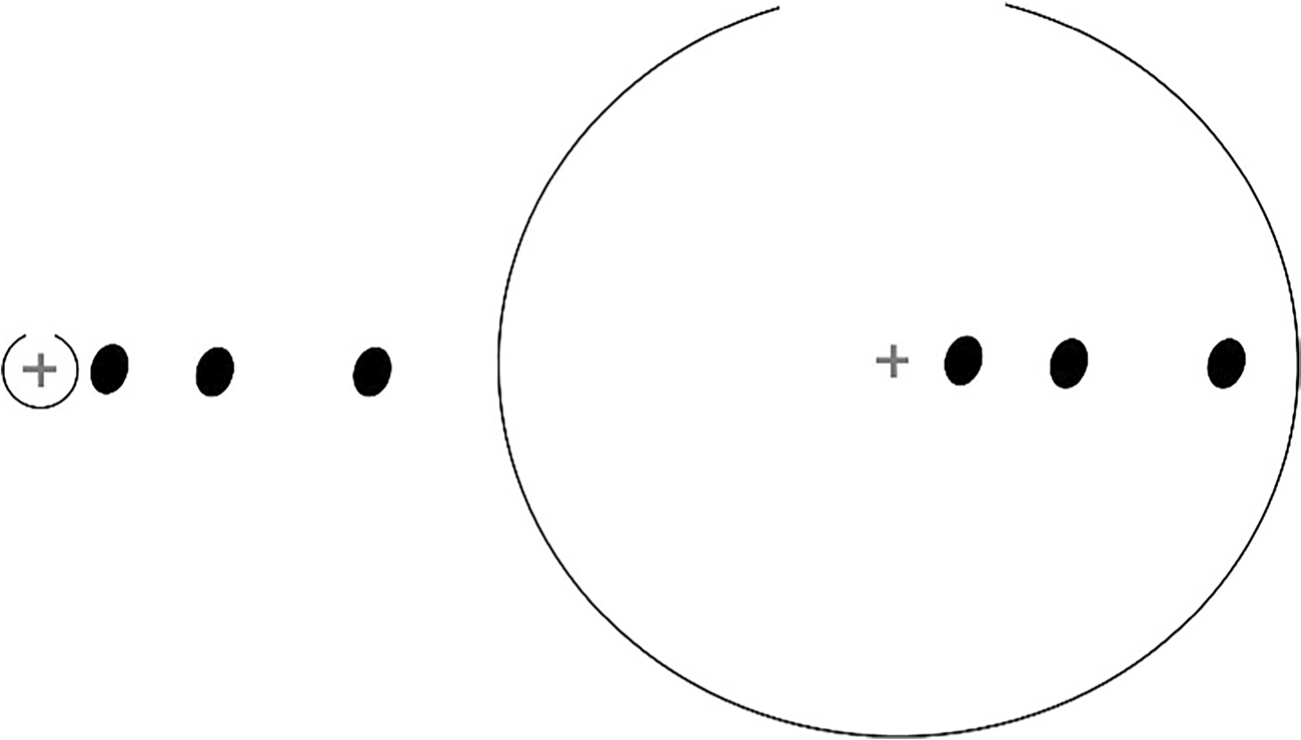
Relative sizes of the stimuli. The target ellipse distance was calculated relative to the centre of the screen, indicated by the fixation cross, which was not visible to the participants

The red colour of the ellipse patches (3.1 cd/m^2^) was a bit darker than the background (6.7 cd/m^2^). The visual search display contained eight ellipses, seven of which were positioned randomly in the central annulus of the screen (radius of 7°), so that they did not overlap with any other ellipses. The long and short axes of each ellipse subtended 0.9° and 0.65° of visual angle, respectively. The minimal allowed distance between the edges of two ellipses was 0.45° of visual angle vertically and 0.33° of visual angle horizontally. One of the ellipses – the target ellipse – was tilted 17.5° to the left or right and was presented at one of three possible distances from the centre of the screen: 1°, 3°, or 6° of visual angle. Figure 2 illustrates the relative proportions of the two circles and the ellipses.

### Pre-processing of behavioural data

To make sure the participants in the final sample were sufficiently engaged in the task, we excluded from further analysis two participants whose response accuracy in at least one of the two experimental tasks was less than 60%. Next, we removed trials with very fast (< 100 ms) or slow (> 3,000 ms) RTs from the ellipse task (*M* = 3.78%). The final sample consisted of 22 participants. The overall sample mean response accuracy was 83.1% in the ellipse task and 91.5% in the circle task.

### Recording and pre-processing of pupil data

The pupil sizes of the left and right eyes were recorded throughout the experiment with a Tobii X120 eye-tracker (Tobii Technology I, 2010) at a rate of 120 Hz. Calibration and validation of gaze location were conducted before the first and fifth blocks of trials.

Prior to analysis, the pupil data of the final sample were pre-processed following the guidelines and MATLAB Toolbox described in Kret and Sjak-Shie (2019). First, blinks detected automatically by the eye-tracker were regarded as missing data. Second, the dilation speed filter detected outliers that were characterized by a sudden change in pupil size (i.e., samples that were disproportionately larger than their adjacent samples). The threshold value to detect speed outliers was defined as the sum of the median dilation speed and its median absolute deviation, with a scaling factor of 32. Third, to remove edge artefacts, all missing data windows were extended in both directions by 50 ms. Fourth, samples that deviated from a smooth trend-line or samples for which pupil diameter was outside of a feasible range (below 1.5 mm or above 9 mm) were removed. Finally, temporally isolated samples were removed from the data (i.e., samples shorter than 50 ms detected before or after missing data sections). Linear interpolation was used to up-sample the data to 1000 Hz and to reconstruct the missing portions of data shorter than 250 ms (Kret & Sjak-Shie, 2019). Finally, a low-pass filter with a cut-off frequency of 4 Hz was used to smooth the signal.

Trials with more than 50% of missing pupil data were excluded from analysis. Each participant in the final sample had more than 50% of pupil data left after the pre-processing and interpolation of the data (average loss 3.3%). To analyse stimulus-related pupil dilations, the average pupil size recorded during the pre-trial baseline period (500 ms prior to stimulus onset) was subtracted from the magnitude of the stimulus-related pupil dilation on that trial.

### Drift diffusion modelling

The drift diffusion analysis used pre-processed response data from the visual search task, but also included the error trials. We used the EZ DDM (Wagenmakers et al., 2007), which decomposes observable task behaviour into three latent decision-making parameters: drift rate (*v*), non-decision time (*Ter*), and boundary separation (*a*). Drift rate reflects the speed of information accumulation for one of the two decision alternatives (target ellipse tilted to the left or the right). Non-decision time reflects the time taken by stimulus encoding and motor execution*.* Boundary separation reflects the amount of information required to reach one of the decisions; the lower the boundary separation, the less evidence is required for committing to a response*.* The EZ diffusion model was fitted to participants’ response time and response accuracy data from the six different task conditions (2 breadth of attention × 3 target ellipse distances from the centre of the screen).

### Statistical analysis

Data were analysed with R (R Core Team, 2014) and RStudio (RStudio Team, 2015) software. DDM parameters, RTs of the correct responses and error rates were analysed with mixed model analysis using the R package lme4 (Bates et al., 2015). The task variables (circle size and target distance) were entered as fixed factors and subjects were treated as a random factor.

Generalised additive mixed models (GAMMs) were used to analyse the pupil time series data using the R packages mgcv (Wood, 2019) and itsadug (van Rij et al., 2020). GAMM analysis allows researchers to model non-linear relationships between the dependent variable and predictor variables. Thus, this approach is especially appropriate for modelling pupil size data that varies nonlinearly with time from stimulus. Additionally, the GAMM analysis enables the detection of the precise onset of the differences between conditions. Moreover, it offers an effective means of controlling for confounding variables, particularly eye vergence and gaze location.

In the GAMM model we tested the effect of task variables on the mean pupil diameter on each trial. The pupil data were down-sampled to 15 Hz to reduce autocorrelations and to make the models computationally less demanding. To control for non-linear trends, two factor smooths were defined in the model. Factor smooths allow the relationship between a dependent and predictor variable to be non-linear by defining the expected value of the dependent variable through a smooth monotonic function of the linear predictor (Wood, 2017). With this approach we were able to take into account dynamic changes in pupil size over the course of the trial. The main model was defined as follows: Pupil ~ Condition + s(Time, by = Condition) + s(Subject, Time, bs = ’fs’, m = 1) + s(right eye x gaze position, right eye y gaze position) + s(divergence), where s() refers to the smooth function. The regression term “Condition” reflects the effect of the experimental condition (narrow vs. broad breadth of attention) after controlling for sources of variance represented by the remaining model terms. Specifically, the first factor smooth in our model was a function of time by condition. The second was a by-subject factor smooth accounting for time. Additionally, to control for the influence of gaze location and eye vergence on pupil size, the X and Y gaze coordinates and the difference between the horizontal gaze positions of the two eyes (right axis X – left axis X) were included in the analysis. The analysis accounted for the hierarchical structure of the data, with time bins nested within trials, which were further nested within subjects.

The analysis of time series data, especially pupil signal, can suffer from highly autocorrelated errors (i.e., the error of one time period is correlated with the subsequent time period error; Baayen et al., 2018; van Rij et al., 2019). This can lead to false discoveries as highly autocorrelated errors increase the probability of Type I error (van Rij et al., 2019). To control whether autocorrelations influenced the results, we tested a model with factor smooths for each unique time series (i.e., combination of participants and trials), which has been shown to effectively reduce autocorrelations. As every participant had up to 384 unique time series, the full model including all the data was computationally too demanding. Thus, to run the model with autocorrelation correction, we chose five blocks of trials (in total 240 trials) randomly from each participant. This model is presented in the Online Supplementary Materials (OSM).

Lastly, we were interested in whether the variance in pupil size mediated the observed effect of breadth of attention on the RTs in the ellipse task. To accomplish this, we conducted causal mediation analyses using the R package mediation with a value of 4,000 simulations per analysis to get consistent estimates (Tingley et al., 2014). We constructed a moderated mediation model to examine whether pupil size mediates the effect of breadth of attention on visual search performance. As a larger pupil may be more beneficial for detecting peripheral targets while a smaller pupil may help to detect centrally presented targets (Mathôt, 2018), we used the target distance from the centre of the screen as a moderator of the effect of pupil size on visual search performance. To focus exclusively on within-subject variance, we used within-subject standardized measures of pupil size as well as reaction time. Standardized pupil size was extracted as the standardized residuals from a GAMM model identical to the main model, but without the condition effects (i.e., the condition fixed effect and the smooth function of time by condition). Next, for each trial, we calculated the mean pupil size at the start of the ellipse task (the first 200 ms of the visual search display), during which there was a difference in pupil size between the breadth-of-attention conditions. Notably, due to the slow nature of the pupil response (Mathot, 2018), the difference in pupil size during this time interval cannot be caused by the visual search display itself but reflects the difference started during the circle task. Thus, the model was defined as follows: the predictor variable was breadth of attention, the mediator was the mean pupil size at the start of the ellipse task, the dependent variable was the RT in the ellipse task, and target distance was entered as a moderator of the pupil-size effect on visual search performance.

## Results

### Circle task

The average accuracy in the circle task was 91.5%. Accuracy in the narrow-breadth-of-attention condition was slightly lower than in the broad-breadth-of-attention condition (90.2% vs. 92.8%; *t*(21) = 2.68, *p* = .01). This suggests that the narrow-breadth-of-attention condition required more mental effort, which may have increased the pupil size in this condition. However, note that this potential confound works *against* the hypothesized direct effect of breadth of attention on pupil size.

### Manipulation check

**Fig. 3:**
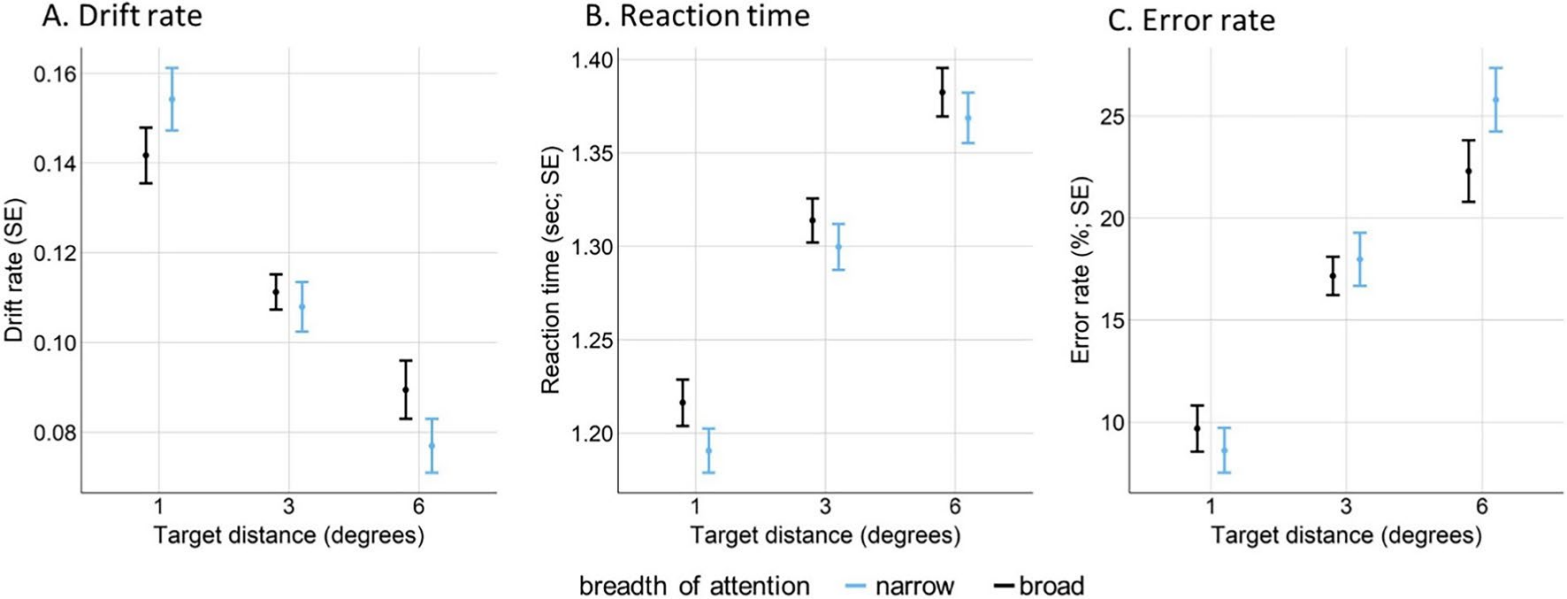
Results of the visual search task

We analysed the DDM parameter drift rate in the visual search task to test our expectation that performance would decrease (indicated by lower drift rate) with increasing target distance from the centre of the screen, and that this decrease in performance would be milder in the broad- compared to narrow-breadth-of-attention condition. A linear mixed effect model was used to assess whether the drift rate parameter was influenced by the breadth-of-attention manipulation (narrow vs. broad) and target distance from the centre of the screen (1°, 3°, 6°). The DDM parameters were calculated for each participant with the EZ DDM algorithm (see Method section). Table 1 and Fig. 3 show the mean drift rates, RTs, and error rates in the ellipse task, separately for each task condition. Inferential statistics for drift rate, RT, and error rate are presented in Table 2. The inferential statistics for the other two DDM parameters (non-decision time and boundary separation) are reported in the OSM.

**Table 1:** Descriptive statistics for the visual search task

		Drift rate		Reaction time (s)		Error rate (%)	
Condition	Target distance	M	SD	M	SD	M	SD
Narrow attention	1°	0.15	0.03	1.19	0.42	8.61	5.16
	3°	0.11	0.03	1.30	0.41	17.98	6.11
	6°	0.08	0.03	1.37	0.43	25.80	7.28
Broad attention	1°	0.14	0.03	1.22	0.43	9.69	5.30
	3°	0.11	0.02	1.31	0.40	17.15	4.39
	6°	0.09	0.03	1.38	0.42	22.30	7.07

Target distance = target ellipse distance from the centre of the screen

**Table 2:** Inferential statistics of drift rate, reaction times, and error rates in the visual search task

*Predictors*	*Estimates*	*SE*	*t*	*df*	*p*
*Drift rate*					
condition [narrow]	0.024	0.013	1.88	107	.06
target distance	-0.026	0.004	-6.25	107	**<.001**
condition [narrow] * target distance	-0.013	0.006	-2.12	107	**.04**
*Reaction time*					
condition [narrow]	-0.03	0.02	-1.28	6777	.20
target distance	0.08	0.01	10.42	6777	**<.001**
condition [narrow] * target distance	0.01	0.01	0.56	6777	.58
*Error rate*					
condition [narrow]	-3.49	2.74	-1.27	107	.21
target distance	6.30	0.90	7.04	107	**<.001**
condition [narrow] * target distance	2.29	1.27	1.81	107	.07

Estimates = unstandardized regression coefficients; *SE* = standard error; *t* = *t*-statistic; *df* = degrees of freedom calculated with Satterthwaite method; *p*-values were calculated with R package lmerTest; *p*-values in bold indicate statistical significance (*p* < .05)

We found that drift rate decreased with an increase in target distance. Importantly, the interaction of breadth of attention and target distance was significant (*p* = .04), indicating that the performance decrement with increasing target distance was less pronounced in the broad-breadth-of-attention condition. These results suggest that the manipulation of breadth of attention was successful. Similar mixed model analyses for correct RTs and error rates only revealed main effects of target distance, with RTs and error rates increasing with the target distance from the centre of the screen (Fig. 3B and C). The interaction between breadth of attention and target distance approached significance for the error rates (*p* = .07). In summary, these results provide evidence for the successful manipulation of breadth of attention.

We performed additional analyses to examine whether the distance between the location of the circle gap and the subsequent target ellipse (gap-to-target distance) influenced the time needed to discriminate that target in the visual search task. As the gap-to-target distances had a different range in breadth of attention conditions, we performed two mixed-model analyses for both conditions where RTs were predicted by the gap-to-ellipse distance. Interestingly, RTs slowed down with increasing gap-to-target distance in the narrow condition (p < .001), but not in the broad condition (p = 0.08). This is consistent with our manipulation of attentional breadth: in the narrow-breadth-of-attention condition targets that were presented far from the gap tended to be outside of the spotlight, resulting in slower RTs, while in the broad condition all of the potential target locations tended to be in the spotlight. Interestingly, these findings also suggest that the focus of the spotlight tended to be drawn toward the location of the gap.

### Pupil time series analysis

After confirming that the attention-breadth manipulation had the expected behavioural effects, we turned to the central question of whether the manipulation also influenced pupil diameter. First, we estimated whether pupil diameter was correlated with divergent eye movements. For each time point we calculated the difference in pupil dilation and eye divergence between the broad- and narrow-breadth-of-attention conditions. The resulting difference scores showed a strong correlation across time points (*r* = .76, *p* < .001), suggesting that pupil dilation was linked to larger eye divergence. Accordingly, and given that pupil size can also be influenced by gaze location (Brisson et al., 2013; Gagl et al., 2011; Hayes & Petrov, 2016; van Rij et al., 2019), we used a GAMM model (see Method) to test the experimental effects on nonlinear time-courses fitted to the pupil data, after controlling for the effects of eye divergence and gaze location.

**Fig. 4:**
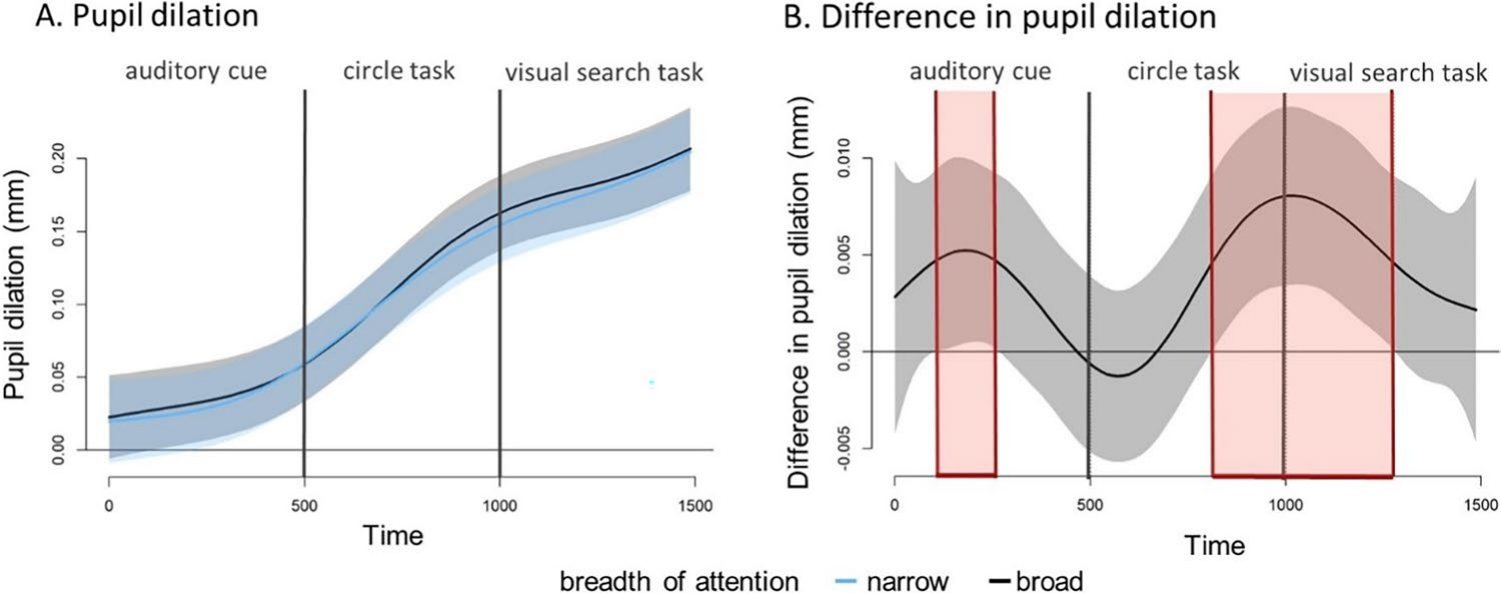
The estimated difference in pupil dilation over time predicted by the generalised additive mixed model model. The error bands represent pointwise 95% confidence intervals. The vertical black line at 500 ms denotes the beginning of the circle task, while the one at 1,000 ms marks the beginning of the ellipse task. **A**. The change in pupil dilation over time per condition. **B**. The difference in pupil dilation between the two breadth-of-attention conditions. The red highlighted area indicates the period with significant differences between the conditions

All the non-linear random effects included in the model were significant (Table 3). These results can be interpreted with the help of Fig. 4. Panel A shows how pupil size develops over time in both experimental conditions, as predicted by the GAMM model. Panel B shows the difference between the narrow- and broad-breadth-of-attention conditions, including the 95% confidence interval. Given that the confidence interval does not include zero during two time periods, the pupil can be said to be significantly more dilated in the broad-breadth-of-attention condition from 106 ms to 256 ms (during processing of the auditory cue) and from 826 ms to 1,277 ms. Note that the second time period started during the breadth-induction procedure and well before the onset of the visual search display. In other words, we found evidence to support the key hypothesis that a broad breadth of attention is associated with a larger pupil size. The model, however, had a large autocorrelation of residuals (*r* = .95). To test whether the high autocorrelations influenced the qualitative pattern of results, we tested a model with factor smooths for each unique time series (22 participants × 240 trials). This model with autocorrelation correction showed similar results for the second time period (see OSM), indicating that the obtained pupil difference between the conditions is valid.

**Table 3:** Results of the generalised additive mixed model analysis

Parametric coefficients	Estimate	*SE*	*t*-value	*p*-value
(Intercept)	0.06	0.01	4.84	**< .001**
Condition narrow	-0.004	0.001	-4.15	**< .001**
*Smooth terms:*	*Edf*	*Ref.df*	*F*	
s(Time):Condition broad	6.57	7.38	37.13	**< .001**
s(Time):Condition narrow	6.12	6.91	37.63	**< .001**
s(X gaze, Y gaze)	21.41	25.59	4.10	**< .001**
s(divergence)	6.74	7.64	189.34	**< .001**
s(Time, Subject)	159.13	197.00	123.32	**< .001**

Deviance explained = 24.5%. Condition broad and narrow refer to the breadth-of-attention conditions in the circle task. Data was down-sampled to 15 Hz prior to analysis. Edf = effective degrees of freedom; Ref.df = reference degrees of freedom; *p*-values in bold indicate statistical significance (*p* < .05)

### Mediation analysis

Lastly, we were interested in whether the within-subject trial-by-trial variance in pupil size mediated the observed effect of breadth of attention on performance in the ellipse task. As it is not possible to compute single-trial drift rates, we focused here on RTs. To examine the average causal mediation effect, we constructed a moderated mediation model with target distance from the centre of the screen moderating the effect of pupil size on visual search (ellipse task). It is important to note that this approach has some limitations as the observed effects in the study were very weak. Specifically, the breadth of attention effect on RTs was not significant, as indicated by the non-significant total effects in the mediation analysis. However, since it has been suggested that in some cases there can be mediation without a significant total effect (O’Rourke & MacKinnon, 2018), we decided to proceed with the analysis. Table 4 shows the results of the mediation analysis for all moderator levels. The mediation analysis showed that the average causal mediation effect (ACME; the indirect effect) of breadth of attention on reaction time via pupil size was not significant.

**Table 4:** Results of the mediation analysis

Moderator level	Type	Effect	Estimate	*p*
target 1°	ACME	breadth of attention -> pupil size -> visual search times	0.002 (-0.001; 0.006)	.18
	direct	breadth of attention -> visual search times	-0.065 (-0.134; 0.004)	.06
	total	breadth of attention -> visual search times	-0.064 (-0.133; 0.006)	.07
target 3°	ACME	breadth of attention -> pupil size -> visual search times	0.001 (-0.0002; 0.003)	.10
	direct	breadth of attention -> visual search times	-0.039 (-0.084; 0.005)	.09
	total	breadth of attention -> visual search times	-0.038 (-0.083; -0.006)	.10
target 6°	ACME	breadth of attention -> pupil size -> visual search times	0.001 (-0.002; 0.004)	.64
	direct	breadth of attention -> visual search times	-0.012 (-0.082; 0.058)	.74
	total	breadth of attention -> visual search times	-0.012 (-0.082; 0.058)	.75

ACME = average causal mediation effect, the indirect effect. The mediation analysis estimates are presented with 95% confidence intervals. breadth of attention = independent variable (circle task), narrow vs. broad condition; visual search times = dependent variable (ellipse task), reaction time in the visual search task

## Discussion

We investigated whether changes in the breadth of attention are accompanied by changes in pupil size, and whether pupil size mediates the behavioural consequences of breadth of attention. To this end, we used trial-wise manipulation of breadth of attention and analysed the dynamic changes in pupil size. The results supported our hypothesis that a broad breadth of attention is associated with a larger pupil size. However, we did not find evidence that pupil size mediated the behavioural effects of the breadth-of-attention manipulation.

Our key finding is in line with previous empirical evidence that changes in attentional breadth coincide with changes in pupil size (Brocher et al., 2018; Daniels et al., 2012; DiCriscio et al., 2018; Eldar et al., 2016; Mathôt & Ivanov, 2019). Importantly, our study did not just replicate the results of these previous studies, but also expanded the set of conditions under which the link between attention breadth and pupil size has been investigated. In addition to presenting the stimuli in the central visual field, we used a trial-wise manipulation of breadth of attention instead of a block-wise approach (DiCriscio et al., 2018), and analysed pupil size changes corresponding with trial-wise shifts of breadth of attention. In addition, unlike several previous studies, we obtained behavioural data which indicated that the manipulation of breadth of attention was successful: A narrow breadth of attention enhanced processing of centrally presented targets and a broad breadth of attention enhanced processing of peripherally presented targets. It is also worth noting that we had some potential effects on pupil size working against our main effect: the narrow breadth-of-attention condition was slightly more difficult for the participants, and we used the high-pitched tone cue for the narrow breadth-of-attention condition (Liao et al., 2016), both of which could have led to stronger pupil dilation in the narrow breadth-of-attention condition. Nevertheless, in line with our expectation, we observed a large dilation for the broad breadth-of-attention condition.

We also sought to minimize the effects of two well-known confounding factors on the relationship between pupil size and breadth of attention: mental effort and the pupil near response. First, the mental effort confound arises from the observation that covertly attending to stimuli presented in peripheral vision requires more mental effort than attending to stimuli presented in central vision (Brocher et al., 2018). Given that higher mental effort has been associated with a dilated pupil, it is possible that prior findings associating broader breadth of attention with larger pupil size are in part driven by increased mental effort. To minimize this confounding effect, we used stimuli that fell broadly within the central visual field (radius of 7°; Strasburger et al., 2011). In addition, we assessed the difficulty of the breadth-induction task by asking participants to remember the gap in the cued circle. Response accuracy was slightly higher for the large circles, suggesting that, if anything, the broad-breadth-of-attention condition required *less* effort.

Second, we addressed problems that are linked to eye movements and measures of pupil size. Using stimuli presented in the central field of view limited convergent and divergent changes in eye positions associated with the pupil near response (Mathôt, 2018), which have been linked to changes in pupil size (Feil et al., 2017). For example, Daniels et al. (2012) showed that in their study the pupil near response contributed to the observed effect of attentional breadth on pupil size. However, compared to our experimental design, in their study the stimuli were presented closer to the participant (38.1 cm; in our study 66 cm), and the stimuli used to induce narrow and broad breadth of attention were wider apart (central stimuli with a radius of 1.3° and peripheral stimuli with a radius of 13°; in our study 0.7° and 7°, respectively). We also quantified the convergence or divergence between the two eyes to assess the possible effect of accommodation movements on pupil size (cf. Daniels et al., 2012). We found that eye divergence had a strong correlation with pupil dilation, suggesting that bigger divergence between the eyes is related to larger pupil dilation, in line with the pupil near response. However, we controlled for eye divergence in the GAMM model and still found a statistically significant effect of breadth of attention on pupil size.

It is possible that we did not entirely remove the potential effects of these confounding variables. However, we showed that the relationship between breadth of attention and pupil size is detectable even if all stimuli are presented in the central field of view, when mental effort and the pupil near response should have a relatively small effect on pupil size. Nevertheless, future studies should focus more systematically on controlling for both these confounds. For example, some studies have tried to match the mental effort required for processing stimuli used in the narrow- versus broad-breadth-of-attention conditions (Mathôt, 2020; Zeng et al., 2017). Controlling for the effect of the pupil near response is more challenging. One problem is that eye divergence is likely a part of the broadening of attention. This means that it might be hard to disentangle the effects of breadth of attention and eye divergence on pupil size. Nevertheless, it is advisable to assess and correct for changes in eye divergence.

In an additional analysis, we did not find evidence that the behavioural effect of breadth of attention was mediated by pupil size. Therefore, while recent work suggests that pupil size directly tunes visual attention (Mathôt, 2020; Mathôt & Ivanov, 2019; Mathôt & Van der Stigchel, 2015), our results do not support the idea that changing pupil size is one of the mechanisms through which attentional control systems achieve the narrowing and broadening of the focus of attention. Instead, it is possible that the neural mechanism regulating breadth of attention also influences the dilation and constriction of the pupil, and hence acts as a “third variable”. As we suggested in the introduction, one of these systems may be the noradrenergic system (Aston-Jones & Cohen, 2005; Joshi & Gold, 2020), which regulates overall neural communication in the brain. However, it is important to note that the mediation analysis conducted to examine this question was limited by the small RT effects observed in our study. Therefore, further research with different manipulations of breadth of attention is necessary to clarify the nature of the relationship between pupil size and breadth of attention.

To conclude, the present study used a novel approach to investigate the relationship between breadth of attention and pupil size. In accordance with previous findings, we found that pupil size scales with the breadth of attention. The functional significance of this relationship remains to be clarified.

### Supplementary Information

Below is the link to the electronic supplementary material.

Supplementary file1 (PDF 176 KB)
