# Supplementary material for: Broadening of attention dilates the pupil
Source: Atten Percept Psychophys. 2023 Oct 6;86(1):146–58. doi: 10.3758/s13414-023-02793-3 (PMC10770199; doi:10.3758/s13414-023-02793-3)
Supplement: Supplementary file 1 — Supplementary file1 (PDF 176 KB) [file 13414_2023_2793_MOESM1_ESM.pdf]

Supplementary Materials

Drift diffusion modelling results in the visual search task

Descriptive statistics of DDM parameters non-decision time and boundary separation are presented in Table S1. Inferential statistics concerning the DDM parameters are presented in Table S2. We conducted two mixed model analyses in which (i) non-decision time and (ii) decision boundary were predicted by breadth of attention and target distance, and participants were entered as a random effect. Both analyses showed only a main effect of target distance.

Table S1. Descriptive statistics of DDM parameters non-decision time and boundary separation.

		Non-decision time		Boundary Separation	
Condition	Target distance	M	SD	M	SD
narrow attention	1°	0.70	0.09	0.18	0.02
	3°	0.83	0.08	0.16	0.02
	6°	0.91	0.09	0.14	0.02
broad attention	1°	0.70	0.07	0.18	0.02
	3°	0.87	0.05	0.15	0.01
	6°	0.92	0.09	0.15	0.02

Note. Target distance = target ellipse distance from the centre of the screen

Table S2. Inferential statistics of DDM parameter non-decision time and boundary separation in the visual search task.

Predictors	*Estimates*	*SE*	*t*	*df*	*p*
Non-decision time					
condition [narrow]	-0.007	0.038	-0.19	107	.85
target distance	0.110	0.013	8.80	107	**<.001**
condition [narrow] * target distance	-0.004	0.018	-0.24	107	.81
Boundary separation					
condition [narrow]	0.002	0.009	0.26	107	.79
target distance	-0.016	0.003	-5.24	107	**<.001**
condition [narrow] * target distance	-0.001	0.004	-0.25	107	.80

Note. Estimates = unstandardized regression coefficients; *SE* = standard error; *t* = *t*-statistic; *df* = degrees of freedom calculated with Satterthwaite method; *p*-values were calculated with R package lmerTest; *p*-values in bold indicate statistical significance ($p < .05$)

GAMM model with autocorrelation correction

The main model was defined as follows: Pupil ~ Condition + s(Time, by=Condition)+ s(right eye x gaze position, right eye y gaze position) + s(divergence) + s(Time, Event, bs="fs"). The first factor smooth in the model was a function of time by condition. The second was the X and Y gaze coordinates, to control for the influence of gaze location on pupil size. The third was the divergence between the right and left eye (right x gaze – left x gaze), to control for the pupil near effect. Finally, we included a random factor smooth for each unique time series. To run the analysis, we selected 5 blocks of trials (240 trials in total) randomly from each participant. Pupil size was significantly larger in the broad-breadth-of-attention condition from 886 ms to 1337 ms (Figure S1). Autocorrelation for this model was -.13, which indicates a small correlation between the residuals, which suggests that autocorrelation is not an issue in the supplementary analysis. Furthermore, our supplementary analysis demonstrated that the main difference between the conditions remains significant even after accounting for the substantial autocorrelation present in the main model. The full analysis script is available in the OSF repository at https://osf.io/h2qsz/.

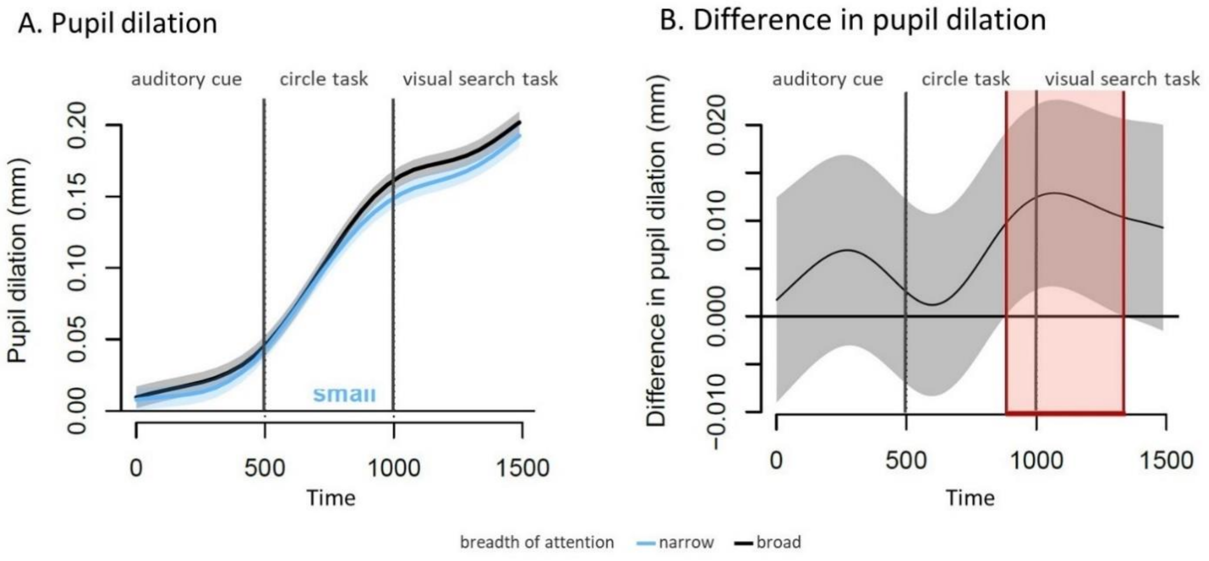

Figure S1. The estimated difference in pupil dilation over time predicted by the GAMM model. The error bands represent pointwise 95% confidence intervals. The vertical black line at 500 ms denotes the beginning of the circle task, while the one at 1000 ms marks the beginning of the ellipse task. A. The change in pupil dilation over time per condition. B. The difference in pupil dilation between the two breadth-of-attention conditions. The red highlighted area indicates the period with significant differences between the conditions.
